# Environmental and behavioral factors associated with household transmission of SARS-CoV-2 in children and adolescents

**DOI:** 10.3389/fped.2023.1239372

**Published:** 2023-10-20

**Authors:** Faétila dos Santos Oliveira, Rafael Alves Guimarães, Eliane Terezinha Afonso, Lusmaia Damaceno Camargo Costa, Karina Machado Siqueira, Solomar Martins Marques, Claci Fátima Weirich Rosso, Paulo Sérgio Sucasas da Costa, Alessandra Vitorino Naghettini, Maria Alves Barbosa, Valéria Pagotto, Natália Del’ Angelo Aredes

**Affiliations:** ^1^ Nursing School, Federal University of Goiás, Goiânia, Brazil; ^2^ Institute of Tropical Pathology and Public Health, Federal University of Goiás, Goiânia, Brazil; ^3^ Medical School, Federal University of Goiás, Goiânia, Brazil

**Keywords:** COVID-19/epidemiology, COVID-19/transmission, household transmission, child, adolescent, risk factors

## Abstract

**Objective:**

To analyze the association between risk behaviors and environmental factors and SARS-CoV-2 infection in children and adolescents in the family environment.

**Methods:**

Cross-sectional study. A total of 267 children and adolescents aged 5–19 years who have contact with COVID-19-positive essential workers were tested between June and October 2020. Behavioral and environmental variables associated with SARS-CoV-2 infection were investigated. Association between these variables was performed using Poisson regression.

**Results:**

SARS-CoV-2 prevalence was 25.1%. Following the confirmation of COVID-19 diagnosis of the index case, 92.1% of adults reported hand hygiene and 83.5% showed habits of respiratory etiquette. However, 12.7% wore masks in common areas of the residence before COVID-19. Sharing common objects was a risk factor for SARS-CoV-2 infection in the sample.

**Conclusion:**

Sharing objects among family members was identified as a risk factor associated with SARS-CoV-2 infection in children and adolescents who lived with infected adults. There was high frequency of hand hygiene and low prevalence of mask use.

## Introduction

As research on the COVID-19 pandemic progresses, special attention has been given to children who currently do not have wide vaccination coverage and, in some countries, there is still debate about vaccinating children under 12 years of age (1), where worsening cases with the Pediatric Multisystem Inflammatory Syndrome (MIS-C) (2) were reported and among whom there is still no clarification about the prevalence and manifestation of Long-term effects of COVID-19 (long COVID) (3).

Data from the United Nations Children's Fund (UNICEF) showed that 17,200 deaths from COVID-19 occurred in children and adolescents aged up to 20 years, with 53% in adolescents aged 10–19 years and 47% in children aged 0–9 years (4). In Brazil, data indicate that among the 23,277 cases of COVID-19 in children and adolescents, 1,449 deaths were recorded in the age group from zero to 11 years since the beginning of the pandemic (5).

In this regard, since 2020, competent health agencies, such as the Centers for Disease Control and Prevention (CDC) and the World Health Organization, have recognized the ways in which SARS-CoV-2 is transmitted and have guided measures to contain viral spread, among them the use of face masks (except for children under two years of age) (6, 7), hand hygiene, respiratory etiquette, social distancing, and isolation of suspected and confirmed cases. The last measure adopted in many countries was vaccination against COVID-19, which in adolescents and children aged 5–11 years is more recent and started in Brazil in 2022, approximately one year after the start of vaccination in adults (8). It should be noted that vaccination does not dispense with the adoption of biosafety behaviors mentioned above, adding to the list of disease prevention actions.

According to the current context, transmission involving children and adolescents can take place both in the home environment and in living spaces, including the school. However, the transmission of the virus essentially in the home environment was, for a long time, a reality observed during the pandemic, when schools suspended in-person activities (9) (measure that, in Brazil, lasted until the end of 2021). Studies show that children and adolescents were mostly infected by an adult in the family group (10–12), confirming the relevance of the topic of household contact in what regards transmission of SARS-CoV-2 (13–16).

There is a gap in the literature worldwide concerning the knowledge of family risk and protective behaviors for transmission of COVID-19 to children and adolescents from an adult index case, especially in the studies analyzing the association between behavioral and environmental variables and the SARS-CoV-2 infection in this group. Thus, the objective was to analyze the association between risk behaviors and environmental factors and SARS-CoV-2 infection in children and adolescents in the family environment.

## Methods

This is a cross-sectional study. The study was carried out in Goiânia, capital of the State of Goiás, Central-West Region of Brazil, from June 15 and October 28, 2020. On the study period, the capital had 1,536,097 inhabitants, of which 298,043 were children and adolescents aged between 5 and 19 years (17–19). The number of confirmed cases of COVID-19 in Goiânia was 3,452 at the beginning of the study (June 2020), with 6% in people under 19 years of age. By the end of the study (October 2020), the total number of COVID-19 cases had increased to 67,871, with 8% being children and adolescents (18, 19).

The study population included children and adolescents aged between 5 and 19 years, who were not hospitalized and who were household contacts of SARS-CoV-2 positive essential workers, confirmed by the real-time quantitative reverse transcription polymerase chain reaction test (RT-qPCR).

We defined household contacts as those children and adolescents who lived with primary COVID-19 cases in a household for at least one day after the index case tested positive for SARS-CoV-2 regardless of whether they were direct family members (19).

As inclusion criteria, the following were adopted: (i) age between 5 and 19 years and (ii) residing in the same household as the index case of COVID-19, regardless of the degree of kinship. There was no restriction on the number of children and adolescents per household and they could or could not be symptomatic at the time of the research (19). Only one index case per household was considered for the study, that is, the first adult diagnosed with SARS-CoV-2. The exclusion criterion adopted was the infeasibility of the biological sample collected for diagnostic examination, which occurred in cases of participant agitation during the procedure (nasopharyngeal sample collection with swab).

All essential workers who had laboratory confirmation for SARS-CoV-2 infection (index case) through a project of the Federal University of Goiás (Central Brazil) were contacted for analysis of SARS-CoV-2 transmission to children and adolescents (target population of this study). Thus, the study had as its starting point adult essential workers with a diagnosis of COVID-19 confirmed by RT-qPCR, and who lived with children in the home environment (19).

The selection of adults who generated the recruitment of children and adolescents took place by non-probabilistic convenience sampling based on the dissemination of the research on social media, television, and radio aimed at workers who at that time were almost the only ones working in the in-person modality, due to the restrictive measures. The research recruited volunteers, public health and safety workers with flu-like symptoms, for COVID-19 testing, nursing and medical consultation, and an initial risk factor interview (19).

Based on the positive cases, the person responsible for voluntary participation in the research was invited to include children and adolescents in the present study. At that moment, clarifications were provided about the study and, after being accepted by telephone, an in-person appointment was made to obtain the consent and assent of the child as part of the ethical processes in scientific research, health consultation with screening, testing of the child or adolescent, and provision of guidelines, with study data collection, between 24 and 48 h from the invitation to participate.

Data were collected by trained professionals and researchers. This group consisted of pediatricians, pediatric medical residents and nurses. Data collection was carried out in two stages: (1) In-person service and structured interview; and (2) Collection of biological material - sample by nasopharyngeal swab to perform RT-qPCR. The samples were kept refrigerated (from +4 to +8°C) and processed between 24 and 72 h (19).

The test results were communicated to those responsible for the participants through phone calls or messages via the WhatsApp application, within 72 working hours, with the report sent signed by the laboratory professional. At the time of contact to disclose the result, guidelines were given to prevent the spread of COVID-19 in positive cases and, above all, on warning signs in case of clinical worsening and possibilities of referral to the network health services. All cases were reported in the national information system for epidemiological surveillance.

This study assumed that SARS-CoV-2 infection occurred at home, considering that restrictive measures were in force for the data collection period. At the time, schools were closed and children and adolescents were, in general, restricted to their homes.

Data were organized and analyzed using the software Statistical Package for the Social Sciences, version 25.0. Methodological procedures to describe the sample and secondary attack rate (SAR) were described in a previously published study (19). Qualitative variables were described as absolute and relative frequencies, and quantitative variables as mean, median, standard deviation, and interquartile range (IQR).

The study adopted SARS-CoV-2 infection as a dependent variable, characterized by a positive result of the qRT-PCR test, categorized as no or yes. The independent variables were the environmental and behavioral factors possibly associated with SARS-CoV-2, as shown in Table 1.

**Table 1:** Independent behavioral and environmental variables.

Prior to COVID-19	Post-COVID-19
Hand hygiene with soap and water - index case	Maintenance of work activities by the index case
Immediate shower when arriving from work - index case	Permanence of the index case in the residence
Respiratory etiquette habit among family members	Isolation of the index case in a separate room
Use of masks at home common areas.	Respiratory etiquette habit among family members
Habit of separating potentially shared objects	Use of masks at home common areas.
	Disinfection of household surfaces and door knobs
	Daily disinfection of common areas
	Hand hygiene with soap and water - index case
	Habit of separating potentially shared objects
Prior and Pos-COVID-19	
Sleep with index case	
Sleep with other children	

The following were also adopted as adjustment covariates of the model: sex (female and male) of the participants and of the index case; age (in years) of participants; race/skin color (white, black/mixed-race or others that included Asian and Native American, due to the small number of observations); family income (BRL); the family group economic classes (A/B [high socioeconomic status] and C/D/E [medium to low socioeconomic status]) according to Brazilian classification for socioeconomic status (18). (A/B/C/D/E); degree of kinship of the index case with the child or adolescent (mother, father and others); housing conditions (number of rooms in the house); number of adults in the house; number of adults positive for COVID-19 in the household; profession of the index case and presence of comobidities in children and adolescents (no and yes).

Considering a missing data rate of less than 15% for most variables, multiple imputation of behavioral and environmental variables was performed using logistic regression (20). For this, the following adjustment variables were adopted in the imputation: sex of the index case, sex of the child/adolescent, economic class, age of the child/adolescent and profession, when observing that these could interfere in the outcome, that is, in the infection by SARS-CoV-2, as well as with risk behaviors.

After coding each of the behavioral and environmental risk factors, the sum of these factors was performed, generating a maximum score of 17 factors present in each individual. This score was categorized as ≤5; 6–10; and >10.

Subsequently, the positive and negative groups for SARS-CoV-2 were compared using Pearson's chi-square test or Fisher's exact test in the bivariate analysis. Next, all variables were included in a Poisson multiple regression model to adjust for potential confounders. The model was adjusted for confounding variables related to the index case. The model was presented as the Adjusted Prevalence Ratio (APR) and the respective 95% confidence interval (95%CI). Statistical significance was verified by the Wald test.

Therefore, the Omnibus test was performed to check the adjustment of the Poisson regression, which did not show statistical significance (*p* = 0.321), indicating good fitting in the model. In all analyses, *p* values < 0.05 were considered statistically significant.

This study was approved by the Research Ethics Committee of the Hospital das Clínicas of the Federal University of Goiás, protocol No. 4.173.690/2020. Verbal assent was obtained from all children and adolescents included in the study, and verbal consent was obtained from the legal guardian of all participants.

## Results

This study included 267 children and adolescents. Of these, 25.1% were positive for SARS-CoV-2. The index cases were mostly female (53.8%), socioeconomic class C/D/E (93.6%), and health workers (68.9%). The median number of adults in the household was 2 (IQR = 1), with 68.5% having two or fewer adults per household, while the median number of rooms was 6 (IQR = 3), with 56.2% households with six or fewer rooms. The mother accounted for 66.6% of the index cases and there was more than one adult positive for SARS-CoV-2 in 33.0% of the sample. The median age of children and adolescents was 11 years (IQR = 8), with 64.8% being adolescents (28.8% between 10 and 14 years and 36.0% between 15 and 19 years). Most (52.3%) were female and 48.3% were brown or black. Of the total, 17.2% had some self-reported comorbidity, the most prevalent being asthma (10.1%).

The descriptive analysis of behaviors and environmental risk factors collected are presented in Table 2.

**Table 2:** Prevalence of risk behaviors and environmental factors potentially associated with SARS-CoV-2 transmission in children and adolescents. Goiânia, Goiás, Brazil, 2020.

Variables	Yes	No
Child/adolescent, *n* (%)		
Sleep with index case	36 (13.5)	231 (86.5)
Sleep with other children	120 (44.9)	147 (55.1)
Prior to COVID-19, *n* (%)		
Hand hygiene with soap and water—index case	243 (91.0)	24 (9.0)
Immediate shower when arriving from work - index case	191 (71.5)	76 (28.5)
Respiratory etiquette habit among family members	206 (77.2)	61 (22.8)
Use of masks at home common areas.	34 (12.7)	233 (87.3)
Habit of separating potentially shared objects	136 (50.9)	131 (49.1)
Post-COVID-19, *n* (%)		
Maintenance of work activities by the index case	22 (8.2)	245 (91.8)
Permanence of the index case in the residence	228 (84.4)	39 (15.6)
Isolation of the index case in a separate room	153 (57.3)	114 (42.7)
Respiratory etiquette habit among family members	223 (83.5)	44 (16.5)
Use of masks at home common areas.	180 (67.4)	87 (32.6)
Disinfection of household surfaces and doorknobs	147 (55.1)	120 (44.9)
Daily disinfection of common areas	158 (59.2)	109 (40.8)
Hand hygiene with soap and water - index case	246 (92.1)	21 (7.9)
Habit of separating potentially shared objects*	200 (74.9)	67 (25.1)

*n *= 267.

* Objects such as sink soap, hand towel, toothpaste.

**Figure 1:**
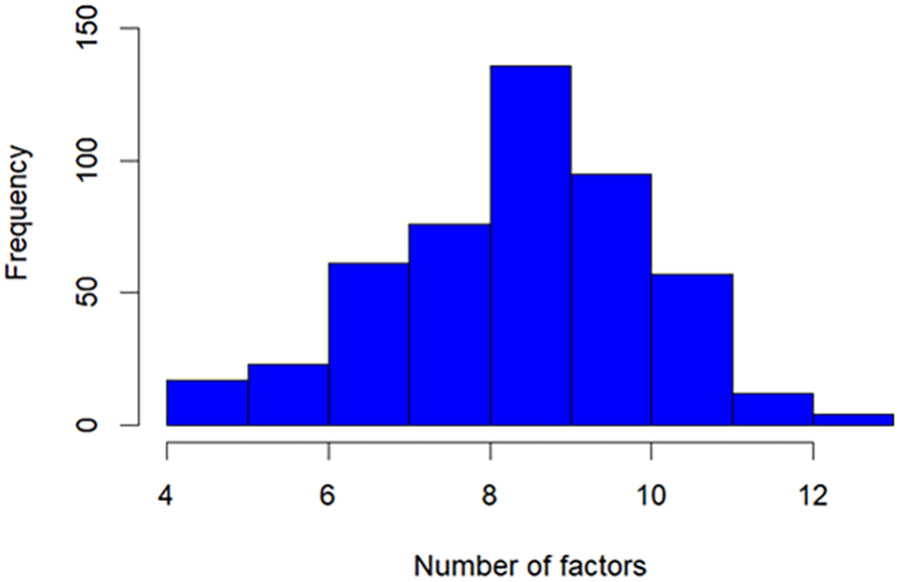
Number of behavioral and occupational risk factors of children and adolescents in contact with essential workers with COVID-19. Goiania, Goiás, Brazil, 2020.

The median number of behavioral and environmental risk factors was 9 (IQR = 2), the mean was 8.8 factors (SD = 1.7), a minimum of 4 and a maximum of 13. The highest frequency was for children and adolescents with 9 factors (28.1%) (Figure 1).

Behaviors and environmental factors were compared in the bivariate analysis, finding no statistically significant difference between groups of children whose tests were positive for SARS-CoV-2 infection vs. negative (*P* value ≥0.05) as shown in Table 3.

**Table 3:** Comparison of the environment of children and adolescents included in the study and behavior of the index case and family group among positive and negative SARS-CoV-2 groups. Goiânia, Goiás, Brazil, 2020.

Variables	SARS-CoV-2 positive (*n* = 67)	SARS-CoV-2 negative (*n* = 200)	*P*-value**
Child/adolescent, *n* (%)			
Sleep with index case	13 (19.4)	31 (15.5)	0.456
Sleep with other children	94 (47.0)	26 (38.8)	0.243
Prior to COVID-19, *n* (%)	–	–	–
Hand hygiene with soap and water - index case	64 (95.5)	179 (89.5)	0.136
Immediate shower when arriving from work - index case	47 (70.1)	144 (72.0)	0.771
Respiratory etiquette habit among family members	48 (71.6)	158 (79.0)	0.214
Use of masks at home common areas.	8 (11.9)	26 (13.0)	0.822
Habit of separating potentially shared objects	29 (43.3)	107 (53.5)	0.148
Post-COVID-19, *n* (%)	–	–	–
Maintenance of work activities by the index case	6 (9.0)	16 (8.0)	0.806
Permanence of the index case in the residence	57 (85.1)	171 (85.5)	0.932
Isolation of the index case in a separate room	36 (53.7)	117 (58.5)	0.495
Respiratory etiquette habit among family members	56 (83.6)	167 (83.5)	0.987
Use of masks at home common areas.	41 (61.2)	139 (69.5)	0.209
Disinfection of household surfaces and door knobs	37 (55.2)	110 (55.0)	0.750
Daily disinfection of common areas	40 (59.7)	118 (59.0)	0.919
Hand hygiene with soap and water - index case	63 (94.0)	183 (91.5)	0.506
Habit of separating potentially shared objects*	49 (73.1)	151 (75.5)	0.699
Number of factors, *n* (%)			
<5	1 (1.5)	10 (5.0)	0.088
6–10	60 (89.5)	159 (79.5)	
>10	6 (9.0)	31 (15.5)	

* Objects such as sink soap, hand towel, toothpaste.

** Pearson's chi-square or Fisher's exact test.

These factors were adjusted in a Poisson multiple regression model for potential confounding variables. The final model showed that the prevalence of SARS-CoV-2 was 1.65 times higher (APR = 1.65; 95%CI = 1.02−2.67) in children and adolescents with index cases who did not have the habit of separating potentially contaminated objects, as shown in Table 4.

**Table 4:** Multiple regression analysis of behavior and physical environment related to SARS-CoV-2 infection in 267 children and adolescents (household contacts of index cases). Goiania, Goiás, Brazil, 2020.

Variables	APR	95.0% CI	β	*P*-value**
Environment and behavior				–
Sleep with index case	0.96	0.57–1.61	0.27	0.890
Before COVID-19				
Hand hygiene with soap and water - index case^c^	2.07	0.64–6.67	0.60	0.224
Immediate shower when arriving from work - index case^c^	0.91	0.56–1.50	0.25	0.721
Respiratory etiquette habit among family members^c^	0.73	0.45–1.18	0.25	0.199
Use of masks at home in common areas.^c^	1.30	0.61–2.77	0.39	0.505
Habit of separating potentially shared objects^c^	1.65	1.02–2.67	0.24	0.040
Post-COVID-19	–	–	–	–
Maintenance of work activities by the index case^b^	1.22	0.51–2.93	0.45	0.651
Permanence of the index case in the residence^b^	0.86	0.47–1.58	0.31	0.629
Isolation of the index case in a separate room^b^	1.10	0.69–1.76	0.24	0.688
Use of masks at home in common areas.^c^	1.37	0.83–2.26	0.26	0.217
Disinfection of household surfaces and door knobs^c^	0.88	0.53–1.45	0.26	0.606
Daily disinfection of common areas^c^	1.07	0.57–2.01	0.32	0.827
Hand hygiene with soap and water - index case^c^	1.03	0.34–3.15	0.57	0.960
Share objects^a^^,^^c^	0.95	0.57–1.59	0.26	0.856

APR, adjusted prevalence ratio; 95.0%CI: 95.0% confidence interval.

* Wald's statistics.

^a^ Objects such as sink soap, hand towel, toothpaste; β = regression coefficient.

^b^ Reference category: no.

^c^ Reference category: yes.

## Discussion

The study showed a high prevalence of risk behaviors for transmission of SARS-CoV-2 to children and adolescents from an index case. In particular, index cases showed low frequencies of mask use in the family environment, habit of separating potentially shared objects, and disinfection of surfaces and doorknobs at home. On the other hand, participants showed high frequencies of adherence to hand hygiene. Absence of the habit of separating potentially shared objects increased the prevalence of SARS-CoV-2 infection among children and adolescents.

Along with the measures to be further discussed in this section, the contact among household members, including children and adolescents, distancing impact the outcomes, as shown in a recent study concluding that when the index case left the household after infection confirmation, there was a 33.3% chance to not transmit to another household member, compared to 15% without any other measure (21).

The study provides undeniable evidence of low frequency of mask use in the home environment, from the suspicion of a positive case to even after diagnostic confirmation, and the wide adherence to the habit of hand hygiene. The two actions described were recommended and widely disseminated by competent national and international health agencies to control the transmission of SARS-CoV-2 (8, 22).

Considering the forms of transmission of SARS-CoV-2, studies have shown that people living in closed environments are exposed to a greater risk of the virus acquisition (15). Research shows that the use of masks significantly reduces this risk (23), especially when performed before the onset of symptoms and when added to other non-pharmacological mitigation measures, such as hand hygiene (24). Therefore, preventive behavioral measures are required in all contexts, especially at home, which is a closed space shared especially with children and adolescents.

Although supposedly oriented towards prevention measures, especially because the sample of index cases consists mostly of health workers in this study, it was observed that the adoption of several preventive measures had low adherence. In addition, most of these workers were professionally active in the in-person modality, therefore having greater exposure to the virus when compared to people working at home. Thus, greater care was expected with their household contacts to reduce transmission, especially considering the closest daily contact between parents and children under 12 years old at home (25, 26).

The use of masks by family members in common areas of the house, in the period before the diagnosis of COVID-19 was low, regardless of the positivity of children and adolescents. Thus, although not statistically significant, we reinforce the non-use of the mask as a risk factor for the transmission of the virus, even before symptoms appear (22). During the pandemic, the use of masks underwent important changes. At first, given the lack of this personal protective equipment on the market, the prioritization of its use by health professionals was recommended. Additionally, there was a lack of scientific evidence on the protective factor of fabric masks, which came to be considered for use by the general population, given the spread of the virus and the absence of surgical and FFP2 masks. Thus, the global recommendation for the use of masks, including those made of fabric, for the general population, was made in June 2020 (27), and then the preference for the use of surgical and FFP2 masks, after the best supply from the industries, and in the face of the most transmissible variants of the virus, such as Omicron, in 2021 (28).

From that moment to the present day, only children under two years of age are out of the recommendation for generalized use, due to the risk of suffocation (29). Other children have been using special-sized masks for better product efficiency, and this personal protective equipment remains an important form of prevention, especially in crowded spaces, or in the face of flu-like symptoms, including among children and adolescents who can transmit the SARS-CoV-2 virus. A recent experimental study concluded that there was significant difference in oxygen saturation among children (minimum 3-years old) using PFF2 masks with or without valve (30), especially during walking and regular play activities, reporting lower percentages of saturation without the valve (30). Therefore, it is important to be attentive for both efficiency of protection and respiratory safety and quality regarding children under masks use.

Regarding hand hygiene, similarity of high adherence was observed among the compared groups. This demonstrates that good hygiene practices disseminated in the media, as well as respiratory etiquette, positively changed people's routine. In this regard, there are two important reflections. One is that at a given moment, when studying the virus behavior in the environment and in the transmissibility process more deeply, it was understood that the most common form of transmission was through droplets and less through indirect contact (29), emphasizing the need to use a mask, as priority, more than hand hygiene. The second reflection is that, considering that there was no difference between the groups, hand hygiene may have been easily interpreted by the population as a preventive measure, but this does not ensure the correct and effective performance of the procedure. It is recognized in the literature that the cleaning length, the product applied, and the coverage of all areas represent an impact on the effectiveness of sanitization, and this information, that goes beyond the sole recommendation to adopt hygiene measures, must reach the population in all areas of health education—including the use of a variety of media.

Regarding behaviors of hand hygiene under the pandemic context with general instruction to reinforce this habit, it is important to notice that it is impacted differently among people from different contexts. This conclusion is based on the differences on hand hygiene adoption data between the participants of this study and a recent publication from Europe, studying household transmission and emphasizing children and adolescents as well (21).

Unlike the high adherence to hand hygiene found in this present work, still in the context of prevention of contamination through indirect contact, there was lower adherence to disinfection of surfaces and door knobs in the families analyzed, even though this practice was initially recommended by health authorities, when analyzing the time of virus viability in fomites (29), and the practice of disinfection, in 2020, was part of the care list recommended to the population to contain the transmission of the virus (22).

To date, no work has been published on the role of exposure of children and adolescents to SARS-CoV-2 transmission at home focused on household behaviors (21). This study shows that 82.0% of the sample was exposed to 6 to 10 risk factors, with a greater sum of factors among positive cases. This finding may contribute to reinforce the guidelines for prevention against COVID-19 at home, through adherence to good behavioral practices ranging from personal hygiene to sanitary habits of family life, protecting children and adolescents from infection.

It was scientifically recognized the priority to avoid droplets transmission of SARS-CoV-2 and the high importance of masks in this matter. Additionally, and despite the impossibility to state causality, a recent study found RNA samples in fomites and hands among negative household members, reporting the risk of infection and reinforcing the need for hygiene measures such as handwashing and surface cleaning, plus physical distancing, and the use of masks (26).

Such measures are necessary not only when dealing with COVID-19, considering that at the beginning of the pandemic there was a major reduction in the number of pediatric patients assisted in emergencies and in the incidence of the main pediatric diseases caused by other viruses, bacteria and atopic conditions related to pollution (31). It reinforces the importance of protective measures to restrict the advance of respiratory diseases among children and adolescents in both regular daily bases as in possible epidemic outbreaks to come (30).

The present study had the cross-sectional character and the non-probabilistic sample, which affect our results in terms of generalization, as its limitations. In addition, data on behaviors and the home environment were self-reported by the guardian who accompanied the participant for care and testing and are subject to memory and forgetfulness bias.

This study provides compelling evidence by showing the magnitude of environmental risk behaviors in the home environment, especially from the perspective of the public of children and adolescents who are in contact with essential workers. The absence of the habit of separating potentially shared objects was associated with SARS-CoV-2 infection in children and adolescents. The understanding of this phenomenon supports family's and health agencies’ decisions regarding the guidelines to be conveyed, since children and adolescents were infected at home, where preventive measures were most neglected.

## Data Availability

The original contributions presented in the study are included in the article/Supplementary Material, further inquiries can be directed to the corresponding author.
